# Waterborne Infectious Diseases Associated with Exposure to Tropical Cyclonic Storms, United States, 1996–2018

**DOI:** 10.3201/eid2908.221906

**Published:** 2023-08

**Authors:** Victoria D. Lynch, Jeffrey Shaman

**Affiliations:** Columbia University, New York, New York, USA

**Keywords:** bacteria, parasites, enteric infections, respiratory infections, waterborne diseases, Cryptosporidium, Giardia, Legionella, Escherichia coli, Salmonella, Shigella, United States

## Abstract

In the United States, tropical cyclones cause destructive flooding that can lead to adverse health outcomes. Storm-driven flooding contaminates environmental, recreational, and drinking water sources, but few studies have examined effects on specific infections over time. We used 23 years of exposure and case data to assess the effects of tropical cyclones on 6 waterborne diseases in a conditional quasi-Poisson model. We separately defined storm exposure for windspeed, rainfall, and proximity to the storm track. Exposure to storm-related rainfall was associated with a 48% (95% CI 27%–69%) increase in Shiga toxin–producing *Escherichia coli* infections 1 week after storms and a 42% (95% CI 22%–62%) in increase Legionnaires’ disease 2 weeks after storms. Cryptosporidiosis cases increased 52% (95% CI 42%–62%) during storm weeks but declined over ensuing weeks. Cyclones are a risk to public health that will likely become more serious with climate change and aging water infrastructure systems.

Tropical cyclones are a seasonal occurrence in the Eastern United States, where they cause widespread destruction and endanger public health (*1*–*3*). Among many storm-related hazards, extreme flooding is a concern because it can lead to the contamination of recreational, irrigation, and drinking water sources (*4*–*6*) and might increase risks for transmission of waterborne infectious diseases (*7*). Elevated case counts and outbreaks have been attributed to individual storms (*8*), but the effect of tropical cyclones on specific waterborne infections has not been evaluated over multiple storm seasons. Understanding waterborne pathogen transmission is a pressing public health challenge because the burden of disease will likely increase in conjunction with an aging population (*9*), deteriorating drinking and wastewater treatment systems (*10*), and increased storm-related flooding due to climate change (*11*).

Bacterial, parasitic, and viral pathogens cause ≈7.15 million cases of waterborne disease annually in the United States (*12*). Infections are typically mild but can lead to life-threatening enteric or respiratory illness for immunocompromised, young, or elderly persons (*13*,*14*). Cyclonic storms drive transmission because floodwater mobilizes pathogens in the environment and inundates water system infrastructure, which causes further contamination through ineffective treatment or sewage overflows (*15*,*16*). After cyclonic storms, high pathogen loads frequently are detected in floodwater (*17*,*18*) and in environmental and drinking water sources (*19**–**21*). Floods also can contaminate irrigation water used on crops (*22*); therefore, flood-driven contamination can influence transmission of pathogens that are predominantly foodborne.

However, contamination does not necessarily lead to transmission; although extreme weather events have been associated with gastrointestinal illness or specific outbreaks (*23**–**25*), some storms have been found to have no effect on incidence of cases (*26*). Those inconsistent associations reflect the relevance of pathogen-specific factors, particularly pathogen biology and primary reservoirs, in determining the effects of storms on transmission. 

Pathogens that form oocysts or are members of biofilm communities persist in environmental waters for weeks, which can increase the likelihood of transmission (*27*,*28*), whereas pathogens that do not persist in the environment might be flushed from waterways by flooding (*29*). Pathogen biology also affects the efficacy of water treatment; in particular, *Cryptosporidium* and *Legionella* are resistant to common decontamination methods (*30*,*31*), whereas *Giardia* is readily removed from water (*32*). 

Cyclonic storms can also lead to different types of contamination depending on the land use and drinking water or sanitation infrastructure of affected regions. Cattle and poultry are the primary reservoirs for several gastrointestinal pathogens, and flooding near livestock production can contaminate drinking water sources with animal waste (*33*). Flooding near livestock production is of particular concern in rural agricultural regions where many persons rely on private wells that are untreated and vulnerable to inundation (*34*). On the other hand, storms in densely populated areas often lead to floodwater contaminated with human sewage (*35*). Urban flooding also can damage water treatment or distribution systems that serve entire cities, leading to large outbreaks (*36*).

The effect of cyclonic storms on waterborne disease also might depend on storm characteristics that determine the extent of flooding and destruction. Storms are generally defined by windspeed and rainfall, factors that are often weakly correlated with each other upon landfall (*37*) and lead to different conditions in affected areas. Slow-moving storms tend to cause greater accumulation of rain and more severe flooding, whereas tropical cyclones with high windspeeds might bring less rain but cause wind-related property or infrastructure destruction (*1*,*38*). Storm type also could dictate disaster management decisions and individual-level responses, such as the ability to comply with evacuation orders. In addition, storm severity influences healthcare-seeking behavior and healthcare infrastructure. Storm-related disruptions might dissuade persons with mild or moderate conditions from seeking care (*39*), whereas catastrophic storms can prevent persons with urgent needs from accessing healthcare systems (*40*).

Storm severity is projected to increase with atmospheric warming, so developing a thorough understanding of storm effects on waterborne diseases could aid climate change adaptation and public health policies. Previous research has largely focused on specific storms and outbreaks or on nonspecific gastrointestinal illness; however, associations over multiple storm seasons have not been thoroughly examined. In this study, we examined the effects of tropical cyclones on waterborne infectious diseases over more than a decade and determined whether those associations varied by pathogen or type of storm exposure. 

## Methods

### Data

#### Case data

We used surveillance data from the National Notifiable Diseases Surveillance System (NNDSS; https://www.cdc.gov/nndss) to identify weekly cases of cryptosporidiosis, giardiasis, Legionnaires’ disease, *Escherichia coli* infections, salmonellosis, and shigellosis during 1996–2018 for each US state. Those infections are caused by parasitic (*Cryptosporidium* and *Giardia*), biofilm-forming bacterial (*Legionella*), and enteric bacterial (*E. coli*, *Salmonella*, *Shigella)* pathogens that can lead to severe gastrointestinal or respiratory illness. Of the 6 *E. coli* strains, NNDSS only tracks Shiga toxin–producing *E. coli* (STEC) infections. 

The data consist of laboratory-confirmed cases from hospitalizations, emergency department visits, and primary care visits that are reported to local health departments and compiled by state health departments to submit to the Centers for Disease Control and Prevention (CDC), which manages the NNDSS and case definitions (Appendix Table 1). We restricted our analyses to the 30 states plus Washington, DC, that experienced >1 tropical cyclone during the study period and to June–November, the months of the Atlantic storm season. We also used US Census data (*9*) to determine county and state populations during the study period.

#### Storm Data

We obtained storm track, windspeed, and rainfall data for tropical cyclones that made landfall in the United States during 1996–2018 from the hurricaneexposure version 0.1.1 and hurricaneexposuredata version 0.1.0 packages in R (R Foundation for Statistical Computing, https://www.r-project.org). For each county, we defined the primary exposure day as the day with the shortest distance between the county center and the storm track. We used storm track and surface windspeed data from the National Hurricane Center’s HURDAT-2 dataset (https://www.nhc.noaa.gov/data) and included maximum and sustained windspeeds on the primary exposure day. We used rainfall data from the North American Land Data Assimilation System 2 (https://ldas.gsfc.nasa.gov/nldas) and included in our dataset the total daily rainfall in each exposed county from 5 days before to 3 days after the primary exposure day. To inform the selection of exposure variables used in the analysis, we assessed correlations among distance, wind, and rainfall variables, including total and daily maximum rainfall.

### Storm Exposure Definition

Informed by the correlation analysis of storm variables, we defined county-level exposure to storms according to total rainfall, maximum sustained windspeed, and distance from the storm track. In the primary analysis, we defined exposure separately for each variable and repeated the analyses using several exposure thresholds. We considered counties exposed when they experienced 50, 75, or 100 mm of total rainfall associated with the storm or were within 500, 250, or 150 km of the storm track. The National Oceanic and Atmospheric Administration categorizes cyclones as tropical storms or hurricanes on the basis of windspeed (https://www.nhc.noaa.gov/climo); consistent with those definitions, we considered counties exposed to tropical storms when maximum sustained windspeeds were >34 knots but <64 knots (gale-force wind on the Beaufort scale) and exposed to hurricanes when maximum sustained windspeeds were >64 knots. We assessed correlations among the exposure thresholds. To determine state-level exposure, we calculated the percent of the state population in exposed counties during storm weeks and classified the state as exposed if 75%, 50%, 25%, 5%, or any (>0%) of the population was exposed; we repeated the analysis for each of those population thresholds.

In the secondary analysis, we combined storm exposure variables to describe categories of cyclonic storms. We categorized storms as high rain–high wind if total rainfall was >100 mm and windspeed was >64 knots; as high rain–low wind if total rainfall was >100 mm and windspeed was >34 but <64 knots; and as low rain–low wind if total rainfall was <100 mm and windspeeds were >34 but <64 knots. We did not include a low rain–high wind category because no storms met that definition. We considered counties exposed to a specific storm type if the storm met both the rainfall and windspeed criteria. Hurricane-force winds are rare and usually affect a small proportion of a state’s population (Appendix Table 2); therefore, we defined state population exposure thresholds only by rainfall exposure, as in the primary analysis. We considered a state exposed to a given storm type if it met the rainfall-based population exposure threshold (e.g., for a 25% population-exposure threshold, >25% of the state’s population had to be exposed to storm-related rainfall) and any of the counties were exposed to the given storm type.

### Statistical Analysis

We modeled the association between exposure to tropical cyclones and case rates by using a conditional quasi-Poisson model (Appendix), which accounted for overdispersion in the case data (*41*). We compared case rates in weeks with and without storms across matched strata based on state and week of the year. That structure addressed potential confounding due to variation among states (i.e., different state policies regarding storm preparedness or case reporting) and controlled for seasonality. We modeled cyclonic storm occurrence as a binary exposure variable and lagged from 0 to 3 weeks to account for the incubation periods of the pathogens and the potential for delays in seeking healthcare after destructive storms. The model included a flexibly adjusted term for year to control for long-term trends that could affect storm exposure or waterborne infectious disease transmission. We used annual state population as an offset to obtain the rate of cases and we modeled case rates for each pathogen separately. We repeated the analysis for all exposure definitions and population exposure thresholds. We used the Bonferroni-Holmes method to adjust 95% CIs for multiple comparisons. Finally, we repeated the method with counties stratified by drinking water source or for rural or urban location (Appendix).

## Results

**Figure 1:**
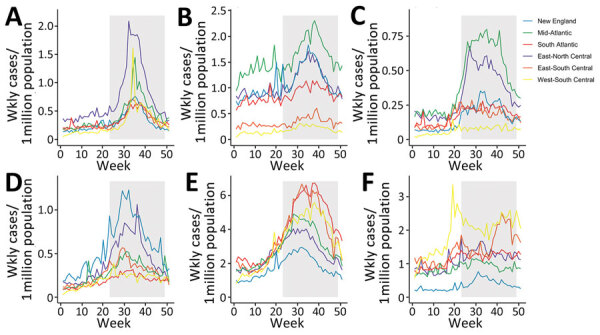
Average weekly cases by geographic region in a study of waterborne infectious diseases associated with exposure to tropical cyclonic storms, United States, 1996–2018. Graphs indicate weekly number of cases per 1,000,000 populations for the following waterborne diseases: A) cryptosporidiosis; B) giardiasis; C) Legionnaires’ disease; D) *Escherichia coli *infection; E) salmonellosis; and F) shigellosis. Not all infections were reported for the entire study period (Table 1). The shaded region represents the weeks encompassed in the annual Atlantic storm season, June 1–November 30. The geographic regions reflect the reporting areas used for infectious disease surveillance in the National Notifiable Diseases Surveillance System (https://www.cdc.gov/nndss). The New England region comprises the states of Connecticut, Maine, Massachusetts, New Hampshire, Rhode Island, and Vermont; the Mid-Atlantic Region comprises New Jersey, New York, and Pennsylvania; the South-Atlantic Region comprises Delaware, Florida, Georgia, Maryland, North Carolina, South Carolina, Virginia, West Virginia, and Washington, DC; the East-North Central Region comprises Illinois, Indiana, Michigan, Ohio, and Wisconsin; the East-South Central Region comprises Alabama, Kentucky, Mississippi, and Tennessee; and the West-South Central Region comprises Arkansas, Louisiana, Oklahoma, and Texas.

The number of cases reported to NNDSS varied by pathogen, and most infections involved enteric bacteria (Table 1). Most infections peaked in the late summer or early fall, but the amplitude of seasonality differed among pathogens and by geographic region (Figure 1). Cryptosporidiosis exhibited the strongest and most consistent seasonality; cases peaked in September in all geographic regions. In most states, Legionnaires’ disease and parasitic infections displayed only a moderate increase during summer months (Appendix Figure 1). Enteric bacterial infections were more common across all states, and salmonellosis showed a strong summer seasonality in most states (Appendix Figure 2). During 1996–2018, Legionnaires’ disease and cryptosporidiosis cases increased, and giardiasis decreased, in all geographic regions; the other infections were relatively consistent over time (Appendix Figure 3). The burden of disease also varied by geographic region; salmonellosis and shigellosis cases were more common in the Southeast, but Legionnaires’ disease was concentrated in the Mid-Atlantic region (Appendix Figure 4). *E. coli* infections, cryptosporidiosis, and giardiasis were all more common in the Upper Midwest and New England states than in other geographic regions (Appendix Figure 4).

**Table 1:** Description of pathogens included in analysis of waterborne infectious diseases associated with exposure to tropical cyclonic storms, United States, 1996–2018*

Pathogen	No. (%) cases in NNDSS	Pathogen type	Incubation period, d (range)†	Estimated cases attributed to waterborne transmission, %‡	Years reported in NNDSS
*Legionella*	77,765 (3.8)	Biofilm-forming bacteria	5–6 (2–10)	97	1996–2018
*Cryptosporidium*	151,573 (7.4)	Parasite	7 (2–12)	43	1998–2018
*Giardia*	297,379 (14.6)	Parasite	7 (1–14)	44	2002–2018
STEC	128,332 (6.3)	Enteric bacteria	0.5–4 (0.5–10)	5	1996–2018
*Salmonella*	964,293 (47.3)	Enteric bacteria	0.5–2 (0.5–16)	6	1998–2016
*Shigella*	421,369 (20.4)	Enteric bacteria	1–3 (0.5–7)	4	1998–2018

*NNDSS, National Notifiable Disease Surveillance System (https://www.cdc.gov/nndss); STEC, Shiga toxin–producing *Escherichia coli*. †According to D.W.K. Acheson (*42*). ‡According to S.A. Collier et al. (*12*).

**Figure 2:**
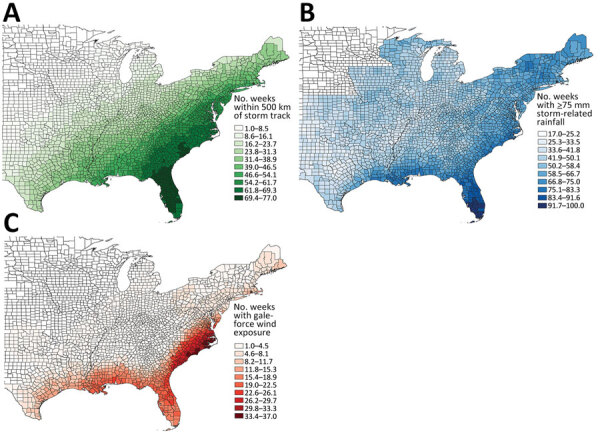
Total number of weeks of storm exposure per county in a study of waterborne infectious diseases associated with exposure to tropical cyclonic storms, United States, 1996–2018. Exposure is defined by 3 factors: A) distance, <500 km of storm track; B) cumulative rainfall of >75 mm; and C) sustained winds above gale-force, i.e., >34 knots.

Wind, rainfall, and distance variables were not highly correlated, but different measures of the same variable, such as maximum rainfall and total rainfall, were correlated (Appendix Figure 4). Among the storm variable thresholds used to determine county-level exposure, hurricane- and gale-force wind exposure were not highly correlated (*r* = 0.21), but >50-mm, >75-mm, and >100-mm rainfall exposure thresholds were highly correlated (*r* = 0.50–0.72) (Appendix Figure 5). Using the most inclusive storm exposure threshold, gale-force wind, 134 cyclonic storms occurred during the study period (Table 2). Those storms affected 2,363 counties in 30 states and Washington, DC, over 177 weeks. Counties with the greatest number of weeks of gale-force wind exposure storms were concentrated along the coast, particularly in North and South Carolina (Figure 2). Exposure to >75 mm of rainfall was most common in South Florida but was overall more widespread and uniform than the wind and distance metrics (Figure 2). We noted no long-term trend in the number of cyclonic storms during the study period (Appendix Figure 6).

**Table 2:** Cyclonic storm exposure definitions used to assess waterborne infectious diseases associated with exposure to tropical cyclonic storms, United States, 1996–2018

Storm exposure variables, definition	No. storms	No. counties affected
Total rainfall, mm		
50	98	2,165	
75	96	2,041	
100	87	1,732
Sustained wind gusts*		
Gale-force winds	134	1,025
Hurricane-force winds	31	136
Distance from storm track, km		
500	134	2,363	
250	134	2,179	
150	117	2,072

*National Oceanic and Atmospheric Administration (https://www.noaa.gov) designates tropical storms as those with gale-force winds, defined as >34 knots to <64 knots, and hurricane-force winds as >64 knots.

**Figure 3:**
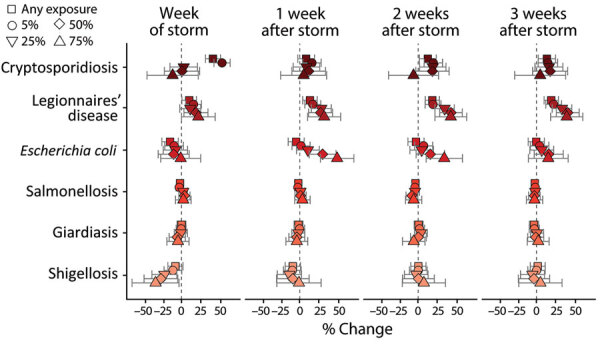
Average percent change in weekly case rates in a study of waterborne infectious diseases associated with exposure to tropical cyclonic storms, United States, 1966–2018. Estimated percentage change (shapes) and Bonferroni-corrected 95% CI (bars) are reported for each infectious disease and population-exposure threshold. Estimates are reported for week of the storm (week 0) and 1–3 weeks after the storm and are associated with exposure to >75 mm of storm-related rainfall.

Cryptosporidiosis case rates greatly increased during storm weeks at low population exposure thresholds; storms that brought >75 mm of rainfall were associated with a 40% increase in case rates when any of the state’s population was exposed and a 52% increase when >5% of the population was exposed (Figure 3). Similar associations persisted across lagged exposures, but the effects were weaker, ranging from 12%–20% increases in the poststorm weeks (Appendix Table 3). Legionnaires’ disease case rates were also highly associated with storm exposure, but the effect was strongest 2 and 3 weeks after a storm and at higher population exposure thresholds (Figure 3). When 75% of the state population was exposed to a storm, case rates increased by 31% in lag week 1, 42% in lag week 2, and 39% in lag week 3 (Appendix Table 3). E. *coli* case rates exhibited a clearer peak and decline associated with lagged storm events. After an initial decrease during the storm week, case rates increased 48% in week 1 and 33% in week 2 post storm when 75% of the state’s population was exposed (Figure 3). Salmonellosis and giardiasis were not greatly associated with storm exposure, and shigellosis case rates slightly decreased during storm weeks (Figure 3).

**Figure 4:**
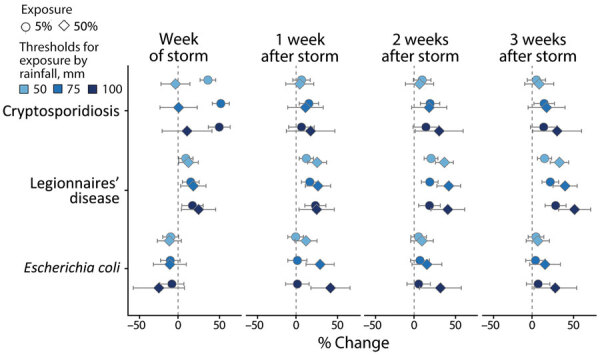
Average percent change in weekly case rates associated with exposure to storm-related rainfall in a study of waterborne infectious diseases associated with exposure to tropical cyclonic storms, United States, 1996–2018. Exposure is defined by 3 cumulative rainfall thresholds, >50 mm, >75 mm, or >100 mm; and for 2 population-exposure thresholds, 5% or 50% exposed. Estimates (shapes) and Bonferroni-corrected 95% CIs (bars) are reported for cryptosporidiosis, Legionnaires’ disease, and *Escherichia coli* infections for the week of the storm (week 0) and 1–3 weeks after the storm.

The associations between storm-related rainfall and cryptosporidiosis, Legionnaires’ disease, and *E. coli* case rates were consistent across different exposure definitions (Figure 4). Storms with less (>50 mm) or more (>100 mm) rainfall were associated with substantial initial increases in cryptosporidiosis cases that attenuated over lag weeks 1–3. The strength of the association between Legionnaires’ disease case rates and storm exposure increased in conjunction with population exposure threshold and amount of rainfall (Figure 4). Similarly, the lagged increase in *E. coli* rates was more pronounced in storms with >100 mm of rainfall. The associations between case rates and storm exposure were similar when exposure was defined by distance from the storm track instead of rainfall (Appendix Figure 7). Stratifying exposure by drinking water source or rural or urban location also yielded similar results; the lagged effect on *E. coli* and Legionnaires’ disease rates was slightly more pronounced when restricted to rural or groundwater-reliant counties, but associations were otherwise consistent (Appendix).

Storm exposure defined by hurricane-force winds was associated with increased cryptosporidiosis case rates 2 and 3 weeks after storms, but otherwise had no effect on rates (Appendix Figure 8). Conversely, gale-force wind exposure was associated with decreased cryptosporidiosis and giardiasis rates during the storm week and had no effect in the lagged weeks after storms (Appendix Figure 8).

**Figure 5:**
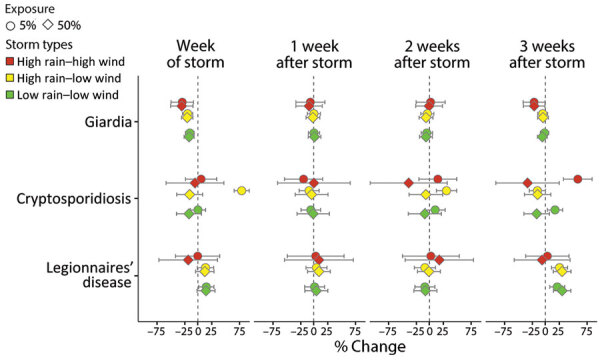
Average percent change in weekly waterborne infectious disease case rates associated with exposure to tropical cyclonic storm types, United States, 1996–2018. Exposure is defined by 3 categories according to rainfall and wind thresholds: high rain–high wind (red); high rain–low wind (yellow); and low rain–low wind (green). Estimates (shapes) and Bonferroni-corrected 95% CIs (bars) are reported for giardiasis, cryptosporidiosis, and Legionnaires’ disease at 2 population-exposure thresholds (shape) for the week of the storm (week 0) and 1–3 weeks after the storm. The population-exposure thresholds refer to the percentage of the state population exposed to storm-related rainfall only; no hurricane-force winds affected >25% of the state population.

Combining wind and rainfall exposure in storm type categories supported the findings of the wind exposure analysis. High rain–high wind, high rain–low wind, and low rain–low wind storms were all associated with decreased giardiasis case rates during the storm week before returning to baseline 1 week poststorm (Figure 5). Consistent with the rainfall analysis, high rain–low wind storms were positively associated with cryptosporidiosis rates up to 2 weeks poststorm, but unlike for rainfall alone, cases also increased 3 weeks after high rain–high wind and low rain–low wind storms: a 58% increase in cryptosporidiosis rates when >5% of the population was exposed to high wind–high rain storms and a 17% increase after low rain–low wind storms (Figure 5).

## Discussion

In this analysis, we found tropical cyclones were associated with waterborne diseases, although the effect magnitude varied by exposure. The associations also differed among the specific pathogens; Legionnaires’ disease, *E. coli*, and cryptosporidiosis rates increased with rainfall, whereas salmonellosis, shigellosis, and giardiasis rates were unaffected, or decreased, during storm weeks. Those divergent associations likely reflect factors that mediate the relationship between storms and disease, including pathogen biology, transmission routes, and severity of infection.

Legionnaires’ disease and *E. coli* case rates consistently increased with rainfall and population exposure thresholds, but the timing of the effects differed between these infections. *E. coli* rates peaked 1 week after storms and returned to baseline by week 3, whereas Legionnaires’ disease rates were highest 3 weeks after storms. Those findings support microbiological studies that have analyzed bacterial counts in streams and water systems after specific hurricanes (*43*,*44*); elevated *E. coli* loads were reported 12–24 hours after a storm started, whereas *Legionella* increased 4–5 days later (*43*). *Legionella* are natural inhabitants of aquatic environments and replicate in water, typically in biofilm communities that colonize household plumbing and water infrastructure systems (*13*,*45*). Thus, the *Legionella* load can increase over time, whereas other bacterial pathogens that do not replicate in the environment typically have bacterial loads that peak after the initial contamination event and dissipate over time (*46*).

Cryptosporidiosis case rates also increased with storm-related rainfall but only at low population thresholds and concurrent with the storm week. Cryptosporidiosis cases were most common in the north-central Midwest, a region that infrequently experiences tropical storms or hurricanes severe enough to affect >25% of the population. The substantial increase in cases concurrent with storm weeks might be driven by several widespread outbreaks attributed to specific storm events that damaged water treatment facilities (*47*). *Cryptosporidium* is resistant to standard chemical disinfectants and is small enough to pass through sand filtration systems common in water treatment plants (*29*); thus, when the parasite contaminates water distribution systems that serve large populations, massive outbreaks can occur (*8*).

County-level exposure to heavy rainfall and cyclonic windspeed often were uncorrelated, which is characteristic of tropical cyclones (*37*), and the effect of extreme wind on cases differed from that of rainfall for several infections. Gale-force wind was associated with a lagged increase in Legionnaire’ disease, but the effect on *E. coli* and cryptosporidiosis was minimal; hurricane-force wind was only associated with increased cryptosporidiosis rates 3 weeks after storms. Such attenuated effects could reflect the intricate, and possibly opposing, factors that influence transmission. High windspeeds are typically associated with destructive storms that can damage sanitation infrastructure, increasing the probability of transmission (*18*), but also could lead to population displacement (*48*), reducing the likelihood that persons will have contact with contaminated water. Extreme storms can also disrupt healthcare systems or alter healthcare-seeking behavior, which can lead to a reduction in detecting or reporting cases (*49*).

For areas that experienced both rainfall and cyclonic wind, we combined exposures into storm-type categories; the results underscored the necessity for pathogen-specific analyses and the limitations inherent in studying events that rarely occur. The high rain–high wind category encompassed the most devastating storms that occurred during the study period, including Hurricanes Katrina and Ivan, but represented a small fraction of all storms. Those events were associated with a substantial decrease in giardiasis but had no effect on Legionnaires’ disease. *Giardia* transmission often occurs in recreational waters, such as swimming pools and rivers, and might be thwarted during storm weeks, when the population is less likely to engage in recreational activities. On the other hand, the burden of Legionnaires’ disease was highest in regions that infrequently experience hurricane-force winds. High rain–high wind storms were associated with a substantial increase in cryptosporidiosis cases 3 weeks after storms, but the effect might have been driven by a 2-month span in 2008 when Texas experienced 2 hurricanes and a third tropical cyclone in succession and reported extremely high cryptosporidiosis case counts for an extended period.

Unlike the other infections, salmonellosis was unaffected by cyclonic storms at all population thresholds. *Salmonella* transmission is predominantly foodborne, and outbreaks attributed to contaminated food are common, particularly during the summer (*42*). The high frequency of salmonellosis outbreaks makes it difficult to detect elevated case counts associated with storms because comparison weeks for storms coincide with those for foodborne outbreaks. Storm-related rainfall was associated with a slight decrease in shigellosis at high population thresholds during storm weeks. Shigellosis is typically mild, and the negative association might reflect a reduction in seeking healthcare for minor illnesses after disruptive storm events.

Except for shigellosis, other disease cases studied exhibited a summer seasonality that coincided with the cyclonic storm season in the United States. However, the inconsistent associations between storms and specific pathogens demonstrated that the effects were not simply driven by overlapping seasonal patterns. Salmonellosis and *E. coli* cases peaked during the same weeks in most regions, but storm-related rainfall had no effect on salmonellosis and a strong positive effect on *E. coli*. This study demonstrated the need for more pathogen-specific analyses that combine microbiological water quality data from multiple sources with epidemiologic data.

One limitation of this study is the spatial mismatch between cases and storm data. Aggregating from county- to state-level storm exposure introduced the possibility of misclassification bias because state-level exposure might be inconsistent with the conditions experienced by cases. We aimed to address this limitation by repeating the analysis at several population thresholds to define exposure and by assessing the consistency of the associations. That type of nondifferential misclassification would also be biased toward the null and underestimate the associations (*50*). Another limitation resulted from the spatial resolution, which only enabled us to perform a rough estimate of the effect of storms stratified by drinking water source or rural or urban location using county-level averages. Highly resolved water source and location data could provide insight into the mechanisms underlying the associations between storms and some waterborne diseases. 

In summary, we found that tropical cyclones represent a risk to public health in the United States, although findings for individual pathogens varied. The US sanitation infrastructure is aging (*10*), and the country will likely experience more severe storm-related flooding as a result of climate change (*11*). Thus, identifying the drivers of pathogen transmission, and opportunities for intervention, will be crucial to reducing disease burden after cyclonic storm events.

Appendix Additional information on waterborne infectious diseases associated with exposure to tropical cyclonic storms, United States.
