# Supplementary material for: Waterborne Infectious Diseases Associated with Exposure to Tropical Cyclonic Storms, United States, 1996–2018
Source: Emerg Infect Dis. 2023 Aug;29(8):1548–58. doi: 10.3201/eid2908.221906 (PMC10370842; doi:10.3201/eid2908.221906)
Supplement: Appendix — Additional information on waterborne infectious diseases associated with exposure to tropical cyclonic storms, United States. [file 22-1906-Techapp-s1.pdf]

Article DOI: https://doi.org/10.3201/eid2908.221906

EID cannot ensure accessibility for supplementary materials supplied by authors. Readers who have difficulty accessing supplementary content should contact the authors for assistance.

Waterborne Infectious Diseases Associated with Exposure to Tropical Cyclonic Storms, United States, 1996–2018

Appendix

Selection of Waterborne Infections and Case Definitions

Case Data and Definitions

The 6 infections in the analysis – Legionnaires' disease, cryptosporidiosis, giardiasis, *Escherichia coli*, salmonellosis, and shigellosis – were selected because they are the only waterborne diseases included in National Notifiable Diseases Surveillance System (NNDSS; https://www.cdc.gov/nndss). The other 11 waterborne illnesses common in the United States, as determined by Centers for Disease Control and Prevention (CDC), are not included in NNDSS. Hospitals, healthcare providers, or laboratories report confirmed cases to public health departments, which notify CDC and send deidentified data to NNDSS (Appendix Table 1).

Supplementary Methods

Data Sources

The storm and case data were the same as described in the main text. Drinking water data were extracted from the Safe Drinking Water Information System (SDWIS; https://www.epa.gov/ground-water-and-drinking-water/safe-drinking-water-information-system-sdwis-federal-reporting), which reports the drinking water source (groundwater or surface water) and population served for each community water system (CWS) in the United States. The primary drinking water source for the county was determined by aggregating the CWSs within the county and calculating the proportion of the population served by groundwater and surface water; counties where $\geq$50% of the population was served by groundwater sources were

categorized as groundwater counties and ≥50% served by surface water as surface water counties. Rurality categories were extracted from the US Department of Agriculture (USDA) Rural-Urban Continuum Codes (https://www.ers.usda.gov/data-products/rural-urban-continuum-codes.aspx), which categorizes metro areas by their population size and nonmetro areas by degree of urbanization and proximity to metro areas. Each county in the study area was classified as urban if it was in a metro category and rural if in a nonmetro category.

Storm Exposure Definition and Statistical Approach

The process for defining state-level exposure was the same as in the main analysis except the counties were stratified by drinking water source and rural or urban location. The population thresholds were any (i.e., >0), 5%, 25%, 50%, and 75% exposed, but only applied to counties that met the drinking water or location criteria. For example, a state was considered exposed to a storm if 25% of the population who lives in rural counties was exposed. We used the conditional quasi-Poisson statistical framework outlined in the main text and Model section (Appendix).

Description of Conditional Quasi-Poisson Model

In the conditional quasi-Poisson framework, the effect of storm exposure is first determined within matched strata created by matching on state and week. For week *i* and state *s*, the within-strata model is:

$$\log\left(\mathrm{E}[Y_{is}]\right) = \alpha_0 + \alpha_{is} + \sum_{l=0}^{4} \beta_l \,\mathrm{storm}_{lis} + ns(\mathrm{year}_s) + \log\left(\mathrm{population}_{is}\right)$$

where α_{is} is the stratum-specific intercept, β_l the lag-specific coefficient (log rate ratio) for storm exposure, storm_{lis} the binary storm exposure variable, $ns(\mathrm{year}_s)$ the spline term for year with two degrees of freedom, and $\mathrm{population}_{is}$ is the population for each state and year. Instead of estimating model parameters, this approach conditions them out by conditioning on the sum of cases for each week *i* in a multinomial model (*1*), such that:

$(Y_{is} \mid \sum_s Y_{is}) \sim \mathrm{Multinomial}\ (\pi_s)$

where:

$$\pi_s = \frac{e^{\boldsymbol{\beta}^T \boldsymbol{x}_s}}{\sum_{j \in i} e^{\boldsymbol{\beta}^T \boldsymbol{x}_j}}$$

The $\beta^T x_s$ and $\beta^T x_j$ terms describe row vectors of coefficients, β, and variables, x, from the quasi-Poisson model where j is the subset of s that includes the observations for each week i.

$$\pi_s = \frac{e^{\alpha_0 + \alpha_i + \sum_{l=0}^{4} \beta_l \text{storm}_{lis} + ns(\text{year}_s) + \log(\text{population}_{is})}}{\sum_{j \in i} e^{\alpha_0 + \alpha_{ij} + \sum_{l=0}^{4} \beta_l \text{storm}_{lij} + ns(\text{year}_j) + \log(\text{population}_{ij})}}$$

Supplementary Results

The effect of storm exposure on cases when exposure was restricted to groundwater counties or rural was essentially the same as the main analysis (Appendix Tables 4, 5). When storm exposure was defined by ≥75 mm rainfall, cryptosporidiosis cases increased during the storm week at low population thresholds but had no significant effect in lagged weeks. Legionnaires' disease cases increased with lagged weeks and higher population exposure thresholds, and the effects were similar to those in the main analysis. At the 50% and 75% population exposure thresholds, the increase in *E. coli* cases 2–3 weeks post storm was more pronounced in groundwater-reliant and rural areas. When exposure was restricted to surface water or rural counties, few states were considered exposed and the effect of storms on most cases was insignificant.

Reference

1. Armstrong BG, Gasparrini A, Tobias A. Conditional Poisson models: a flexible alternative to conditional logistic case cross-over analysis. BMC Med Res Methodol. 2014;14:122. PubMed https://doi.org/10.1186/1471-2288-14-122

Appendix Table 1. Surveillance case definitions used to study waterborne infectious diseases associated with exposure to tropical cyclonic storms, United States, 1996–2018*

Disease	Confirmed case definition	Laboratory criteria for diagnosis
Cryptosporidiosis	A case that meets the clinical description and at least one of the criteria for laboratory-confirmation. When available, species designation and molecular characterization should be reported.	Laboratory-confirmed cryptosporidiosis shall be defined as the detection of a member of the genus Cryptosporidium by one of the following methods:<br>• Organisms in stool, intestinal fluid, or tissue samples, or biopsy specimens<br>• Antigens in stool or intestinal fluid<br>• Nucleic acid by PCR in stool, intestinal fluid, or tissue samples or biopsy specimens
Legionnaires' disease	A clinically compatible case that meets at least one of the confirmatory laboratory criteria.	• By culture: isolation of any *Legionella* organism from respiratory secretions, lung tissue, pleural fluid, or other normally sterile fluid<br>• By detection of *Legionella pneumophilia* serogroup 1 antigen in urine using validate reagents<br>• By seroconversion: 4-fold or greater rise in specific serum antibody titer to *Legionella pneumophilia* serogroup 1 using validated reagents
Escherichia coli infection	A case that meets the laboratory criteria for diagnosis. When available, O and H antigen serotype characterization should be reported.	Isolation of Shiga toxin–producing *Escherichia coli* from a clinical specimen. *Escherichia coli* O157:H7 isolates may be assumed to be Shiga toxin–producing. For all other *E. coli* isolates, Shiga toxin production or the presence of Shiga toxin genes must be determined to be considered STEC.
Salmonellosis	A case that meets the laboratory criteria for diagnosis. When available, O and H antigen serotype characterization should be reported.	Isolation of *Salmonella* from a clinical specimen.
Giardiasis	A case that meets the clinical description and the criteria for laboratory confirmation. When available, molecular characterization (e.g., assemblage designation) should be reported.	Laboratory-confirmed giardiasis shall be defined as the detection of *Giardia* organisms, antigen, or DNA in stool, intestinal fluid, tissue samples, biopsy specimens, or other biologic sample.
Shigellosis	A case that meets the laboratory criteria for diagnosis. When available, O and H antigen serotype characterization should be reported.	Isolation of *Shigella* from a clinical specimen.

*Definitions used by Centers for Disease Control and Prevention for National Disease Surveillance System (https://www.cdc.gov/ndss). STEC, Shiga toxin–producing *Escherichia coli*.

Appendix Table 2. Total number of storm weeks by population- and exposure-threshold across all states in a study waterborne infectious diseases associated with exposure to tropical cyclonic storms, United States, 1996–2018*

	% Population exposed				
Storm exposure variable	Any	5	25	50	75
Total rainfall, mm					
50	4,453	3,734	2,502	1,751	1,100
75	3,199	2,487	1,481	902	476
100	2,210	1,580	865	547	213
Sustained winds†					
Gale-force winds	1,437	1,070	586	284	163
Hurricane-force winds	215	110	6	0	0
Distance from storm track, km					
500	9,108	8,461	7,167	6,107	5,123
250	5,219	4,584	3,400	2,577	1,737
150	3,758	3,151	2,105	1,304	769

*Analysis includes 20,442 weeks, given 27 weeks in the storm season, 30 states and Washington, DC in the affected region, and 23 years of data; West Virginia did not report to the National Notifiable Diseases Surveillance System (NNDSS; https://www.cdc.gov/nndss) in 1996 or 1997.
†National Oceanic and Atmospheric Administration (https://www.noaa.gov) designates tropical storms as those with gale-force winds, defined as $\geq$34 knots to <64 knots and hurricane-force winds as $\geq$64 knots.

Appendix Table 3. Percent change in weekly case rates during weeks with cyclonic storms compared with weeks without storms, United States, 1996–2018*

Disease	% Population exposed	% Change per week post storm (95% CI)			
		Week 0	Week 1	Week 2	Week 3
Cryptosporidiosis					
	>0	0.4 (0.31–0.5)	0.08 (−0.02 to 0.19)	0.12 (0.02–0.23)	0.13 (0.02–0.24)
	5	0.52 (0.42–0.62)	0.16 (0.04–0.27)	0.19 (0.08–0.31)	0.15 (0.02–0.27)
	25	0.03 (−0.14 to 0.2)	0.09 (−0.08 to 0.25)	0.19 (0.02–0.35)	0.16 (−0.01 to 0.33)
	50	0.01 (−0.22 to 0.23)	0.11 (−0.1 to 0.33)	0.18 (−0.03 to 0.39)	0.18 (−0.04 to 0.4)
	75	−0.11 (−0.43 to 0.21)	0.05 (−0.24 to 0.34)	−0.06 (−0.37 to 0.25)	0.05 (−0.27 to 0.37)
Legionnaires' disease	>0	0.1 (0.01–0.19)	0.13 (0.04–0.22)	0.18 (0.09–0.27)	0.19 (0.1, 0.28)
	5	0.15 (0.05–0.25)	0.17 (0.07–0.27)	0.19 (0.09–0.29)	0.22 (0.12–0.32)
	25	0.11 (−0.02 to 0.24)	0.28 (0.15–0.4)	0.34 (0.22–0.46)	0.33 (0.2–0.45)
	50	0.19 (0.03–0.34)	0.27 (0.12–0.42)	0.42 (0.28–0.56)	0.4 (0.26–0.54)
	75	0.22 (0–0.43)	0.31 (0.1–0.52)	0.42 (0.22–0.62)	0.39 (0.19–0.59)
Escherichia coli infection					
	>0	−0.15 (−0.25 to −0.04)	−0.05 (−0.15 to 0.05)	−0.03 (−0.13 to 0.07)	0 (−0.1 to 0.1)
	5	−0.09 (−0.21 to 0.02)	0.02 (−0.1 to 0.13)	0.07 (−0.04 to 0.18)	0.04 (−0.07 to 0.16)
	25	−0.08 (−0.23 to 0.07)	0.1 (−0.04 to 0.25)	0.05 (−0.1 to 0.19)	0.07 (−0.08 to 0.22)
	50	−0.1 (−0.3 to 0.1)	0.29 (0.12–0.46)	0.16 (−0.02 to 0.33)	0.16 (−0.03 to 0.34)
	75	−0.01 (−0.26, 0.24)	0.48 (0.27–0.69)	0.33 (0.11–0.56)	0.15 (−0.1 to 0.4)
Salmonellosis					
	>0	−0.02 (−0.05 to 0.02)	−0.02 (−0.05 to 0.02)	−0.03 (−0.07 to 0)	−0.02 (−0.06 to 0.02)
	5	−0.03 (−0.07 to 0.01)	−0.03 (−0.07 to 0.02)	−0.04 (−0.08 to 0)	−0.02 (−0.06 to 0.03)
	25	0.02 (−0.03 to 0.08)	0.01 (−0.04 to 0.06)	−0.04 (−0.09 to 0.02)	−0.02 (−0.07 to 0.04)
	50	0.04 (−0.03 to 0.11)	0.01 (−0.06 to 0.08)	−0.08 (−0.15 to −0.01)	−0.03 (−0.1 to 0.05)
	75	0.02 (−0.07 to 0.12)	0.03 (−0.06 to 0.13)	−0.06 (−0.16 to 0.05)	−0.02 (−0.13 to 0.08)
Giardiasis					
	>0	−0.02 (−0.05 to 0.02)	−0.02 (−0.05 to 0.02)	−0.03 (−0.07 to 0)	−0.02 (−0.06 to 0.02)
	5	−0.03 (−0.07 to 0.01)	−0.03 (−0.07 to 0.02)	−0.04 (−0.08 to 0)	−0.02 (−0.06 to 0.03)
	25	0.02 (−0.03 to 0.08)	0.01 (−0.04 to 0.06)	−0.04 (−0.09 to 0.02)	−0.02 (−0.07 to 0.04)
	50	0.04 (−0.03 to 0.11)	0.01 (−0.06 to 0.08)	−0.08 (−0.15 to −0.01)	−0.03 (−0.1 to 0.05)
	75	0.02 (−0.07 to 0.12)	0.03 (−0.06 to 0.13)	−0.06 (−0.16 to 0.05)	−0.02 (−0.13 to 0.08)
Shigellosis					
	>0	−0.02 (−0.05 to 0.02)	−0.02 (−0.05 to 0.02)	−0.03 (−0.07 to 0)	−0.02 (−0.06 to 0.02)
	5	−0.03 (−0.07 to 0.01)	−0.03 (−0.07 to 0.02)	−0.04 (−0.08 to 0)	−0.02 (−0.06 to 0.03)
	25	0.02 (−0.03 to 0.08)	0.01 (−0.04, 0.06)	−0.04 (−0.09 to 0.02)	−0.02 (−0.07 to 0.04)
	50	0.04 (−0.03 to 0.11)	0.01 (−0.06 to 0.08)	−0.08 (−0.15 to −0.01)	−0.03 (−0.1 to 0.05)
	75	0.02 (−0.07 to 0.12)	0.03 (−0.06 to 0.13)	−0.06 (−0.16 to 0.05)	−0.02 (−0.13 to 0.08)

*Tropical cyclonic exposure is defined by ≥75 mm cumulative rainfall attributed to the storm.

Appendix Table 4. Effect of exposure in counties with groundwater drinking water sources in a study waterborne infectious diseases associated with exposure to tropical cyclonic storms, United States, 1996–2018*

Disease	% Population exposed	% Change per week post storm (95% CI)			
		Week 0	Week 1	Week 2	Week 3
Cryptosporidiosis					
	>0	0.42 (0.32–0.51)	0.08 (−0.02 to 0.19)	0.12 (0.02–0.23)	0.13 (0.02–0.24)
	5	0.52 (0.41–0.62)	0.13 (0.01–0.25)	0.2 (0.08–0.31)	0.15 (0.03–0.27)
	25	0.03 (−0.15 to 0.2)	0.08 (−0.09 to 0.25)	0.2 (0.03–0.37)	0.16 (−0.01 to 0.34)
	50	0.02 (−0.21 to 0.26)	0.08 (−0.14 to 0.31)	0.15 (−0.07 to 0.38)	0.2 (−0.03 to 0.43)
	75	−0.05 (−0.41 to 0.3)	0.07 (−0.26 to 0.39)	−0.01 (−0.34 to 0.33)	0.12 (−0.23 to 0.46)
Legionnaires' disease					
	>0	0.1 (0.01–0.19)	0.14 (0.05–0.23)	0.18 (0.09–0.27)	0.19 (0.1–0.28)
	5	0.13 (0.03–0.24)	0.17 (0.07–0.27)	0.18 (0.08–0.28)	0.22 (0.12–0.32)
	25	0.11 (−0.03 to 0.24)	0.27 (0.15–0.4)	0.35 (0.22–0.47)	0.35 (0.22–0.47)
	50	0.17 (0.01–0.33)	0.28 (0.12–0.44)	0.38 (0.23–0.54)	0.41 (0.26–0.56)
	75	0.23 (0–0.46)	0.33 (0.1–0.55)	0.38 (0.16–0.61)	0.34 (0.12–0.57)
Escherichia coli infections					
	>0	−0.15 (−0.25 to −0.04)	−0.05 (−0.15 to 0.05)	−0.02 (−0.12 to 0.08)	−0.01 (−0.11 to 0.1)
	5	−0.1 (−0.22 to 0.01)	0.02 (−0.1 to 0.13)	0.06 (−0.05 to 0.18)	0.05 (−0.07 to 0.16)

Disease	% Population exposed	% Change per week post storm (95% CI)			
		Week 0	Week 1	Week 2	Week 3
	25	−0.08 (−0.23 to 0.08)	0.12 (−0.03 to 0.26)	0.04 (−0.11 to 0.2)	0.09 (−0.06 to 0.24)
	50	−0.1 (−0.31 to 0.1)	0.28 (0.1–0.45)	0.17 (−0.01 to 0.36)	0.2 (0.01–0.39)
	75	−0.03 (−0.3 to 0.24)	0.5 (0.28–0.72)	0.39 (0.15–0.63)	0.27 (0.01–0.53)
Salmonellosis					
	>0	−0.02 (−0.06 to 0.02)	−0.02 (−0.06 to 0.02)	−0.04 (−0.07 to 0)	−0.02 (−0.06 to 0.01)
	5	−0.02 (−0.07 to 0.02)	−0.03 (−0.07 to 0.02)	−0.05 (−0.09 to 0)	−0.02 (−0.06 to 0.03)
	25	0.03 (−0.03 to 0.08)	0.02 (−0.03 to 0.07)	−0.03 (−0.09 to 0.02)	−0.01 (−0.06 to 0.05)
	50	0.04 (−0.03 to 0.12)	0.02 (−0.05 to 0.09)	−0.07 (−0.15 to 0)	−0.03 (−0.11 to 0.05)
	75	0.05 (−0.06 to 0.15)	0.06 (−0.04 to 0.17)	−0.04 (−0.16 to 0.07)	−0.02 (−0.13 to 0.1)
Giardiasis					
	>0	0 (−0.06 to 0.05)	−0.01 (−0.07 to 0.04)	0 (−0.05 to 0.06)	−0.03 (−0.09 to 0.03)
	5	−0.01 (−0.07 to 0.06)	−0.01 (−0.07 to 0.06)	0.02 (−0.05 to 0.08)	−0.03 (−0.09 to 0.04)
	25	−0.02 (−0.1 to 0.07)	−0.02 (−0.1 to 0.07)	0.04 (−0.05 to 0.12)	0.01 (−0.08 to 0.09)
	50	−0.05 (−0.16 to 0.06)	−0.04 (−0.15 to 0.06)	0.01 (−0.1 to 0.11)	0 (−0.1, 0.1)
	75	−0.02 (−0.17 to 0.14)	−0.06 (−0.21 to 0.1)	−0.03 (−0.19 to 0.12)	0 (−0.15 to 0.16)
Shigellosis					
	>0	−0.08 (−0.18 to 0.02)	−0.09 (−0.18 to 0.01)	0.01 (−0.09 to 0.1)	0.02 (−0.08 to 0.11)
	5	−0.11 (−0.22 to 0.01)	−0.08 (−0.2 to 0.03)	0.01 (−0.1 to 0.12)	0.01 (−0.1 to 0.12)
	25	−0.19 (−0.35 to −0.03)	−0.11 (−0.26 to 0.05)	−0.01 (−0.16 to 0.14)	−0.03 (−0.19 to 0.12)
	50	−0.26 (−0.48 to −0.04)	−0.07 (−0.28 to 0.14)	0.02 (−0.18 to 0.23)	−0.01 (−0.21 to 0.19)
	75	−0.35 (−0.67 to −0.02)	−0.04 (−0.35 to 0.27)	0.11 (−0.18 to 0.41)	0.09 (−0.2 to 0.39)

*Effects were limited to populations exposed to tropical cyclonic storms in counties with groundwater drinking water sources. Tropical cyclonic exposure is defined by ≥75 mm cumulative rainfall attributed to the storm.

Appendix Table 5. Effect of exposure to rural counties in a study waterborne infectious diseases associated with exposure to tropical cyclonic storms, United States, 1996–2018*

Disease	% Population exposed	% Change per week post storm (95% CI)			
		Week 0	Week 1	Week 2	Week 3
Cryptosporidiosis					
	>0	0.44 (0.34–0.54)	0.1 (−0.01 to 0.21)	0.16 (0.05–0.26)	0.16 (0.05–0.28)
	5	0.56 (0.46–0.66)	0.17 (0.05–0.29)	0.24 (0.12–0.35)	0.18 (0.05–0.3)
	25	0.03 (−0.15 to 0.21)	0.09 (−0.09 to 0.26)	0.22 (0.05–0.39)	0.17 (−0.01 to 0.35)
	50	0.06 (−0.18 to 0.29)	0.16 (−0.06 to 0.39)	0.24 (0.01–0.46)	0.23 (0–0.47)
	75	−0.42 (−0.89 to 0.05)	−0.31 (−0.73 to 0.11)	−0.41 (−0.88 to 0.05)	−0.22 (−0.67 to 0.24)
Legionnaires' disease					
	>0	0.1 (0.01–0.2)	0.14 (0.05–0.23)	0.17 (0.08–0.26)	0.2 (0.1–0.29)
	5	0.15 (0.04–0.25)	0.18 (0.07–0.28)	0.19 (0.09–0.29)	0.23 (0.13–0.34)
	25	0.13 (0–0.26)	0.3 (0.18–0.43)	0.37 (0.24-0.49)	0.34 (0.22–0.47)
	50	0.21 (0.05–0.37)	0.27 (0.11–0.44)	0.42 (0.27-0.57)	0.4 (0.25–0.56)
	75	0.28 (0.05–0.51)	0.3 (0.07-0.54)	0.41 (0.18–0.63)	0.45 (0.23–0.67)
Escherichia coli infection					
	>0	−0.13 (−0.24 to −0.03)	−0.03 (−0.14 to 0.07)	0.01 (−0.09 to 0.11)	0.02 (−0.09 to 0.12)
	5	−0.1 (−0.22 to 0.02)	0.02 (−0.1 to 0.14)	0.07 (−0.04 to 0.19)	0.07 (−0.05 to 0.19)
	25	−0.08 (−0.24 to 0.08)	0.13 (−0.02 to 0.28)	0.06 (−0.09 to 0.22)	0.11 (−0.05 to 0.26)
	50	−0.11 (−0.32 to 0.1)	0.33 (0.15, 0.51)	0.22 (0.03, 0.41)	0.11 (−0.1 to 0.31)
	75	0 (−0.3 to 0.3)	0.51 (0.26, 0.77)	0.49 (0.22, 0.75)	0.23 (−0.07 to 0.53)
Salmonellosis					
	>0	−0.02 (−0.05 to 0.02)	−0.02 (−0.05 to 0.02)	−0.03 (−0.07 to 0)	−0.02 (−0.06 to 0.01)
	5	−0.02 (−0.06 to 0.03)	−0.03 (−0.07, 0.01)	−0.06 (−0.11 to −0.02)	−0.02 (−0.06 to 0.03)
	25	0.03 (−0.02 to 0.09)	0.01 (−0.05 to 0.07)	−0.03 (−0.09 to 0.02)	0 (−0.06 to 0.06)
	50	0.03 (−0.05 to 0.1)	−0.02 (−0.1 to 0.06)	−0.09 (−0.17 to 0)	−0.02 (−0.1 to 0.06)
	75	0.05 (−0.08 to 0.17)	−0.02 (−0.15 to 0.1)	−0.1 (−0.24 to 0.03)	−0.01 (−0.14 to 0.12)

*Effects were limited to populations in rural counties exposed to tropical cyclonic storms, defined by ≥75 mm cumulative rainfall attributed to the storm.

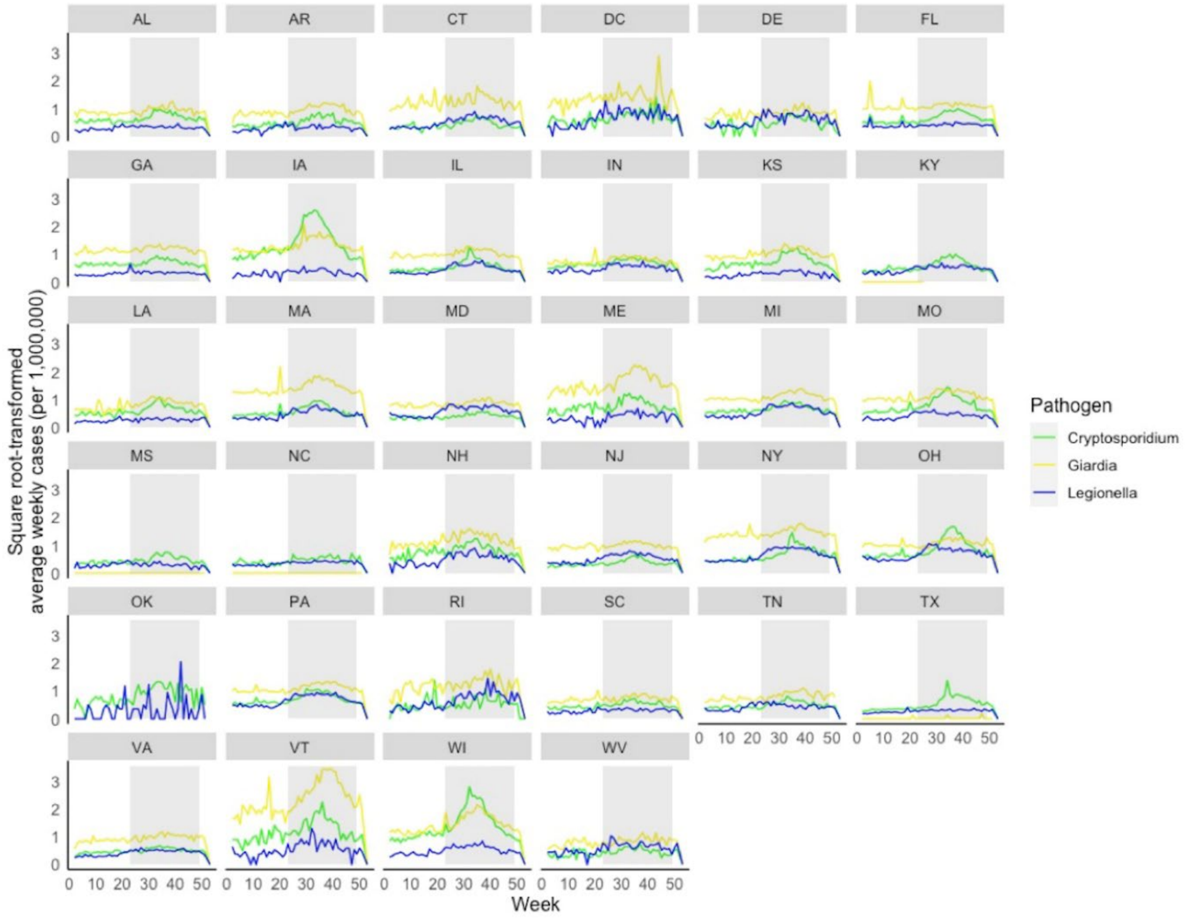

Appendix Figure 1. Average weekly cryptosporidiosis, giardiasis, and Legionnaires' disease cases per 1,000,000 population by state in a study of waterborne infectious diseases associated with exposure to tropical cyclonic storms, United States, 1996–2018. Average weekly cases were square-root transformed for infectious disease surveillance data in the National Notifiable Diseases Surveillance System (https://www.cdc.gov/nndss); not all infections were reported for the entire study period (Table 1). The shaded region in each graph represents the weeks encompassed in the annual Atlantic storm season, June 1–November 30. AL, Alabama; AR, Arkansas; CT, Connecticut; DC, Washington, DC; DE, Delaware; FL, Florida; GA, Georgia; IL, Illinois; IN, Indiana; KY, Kentucky; LA, Louisiana; MA, Massachusetts; MD, Maryland; ME, Maine; MI, Michigan; MS, Mississippi; NC, North Carolina; NH, New Hampshire; NJ, New Jersey: NY, New York; OH, Ohio; OK, Oklahoma; PA, Pennsylvania; RI, Rhode Island; SC, South Carolina; TN, Tennessee; TX, Texas; VA, Virginia; VT, Vermont; WI, Wisconsin; WV, West Virginia.

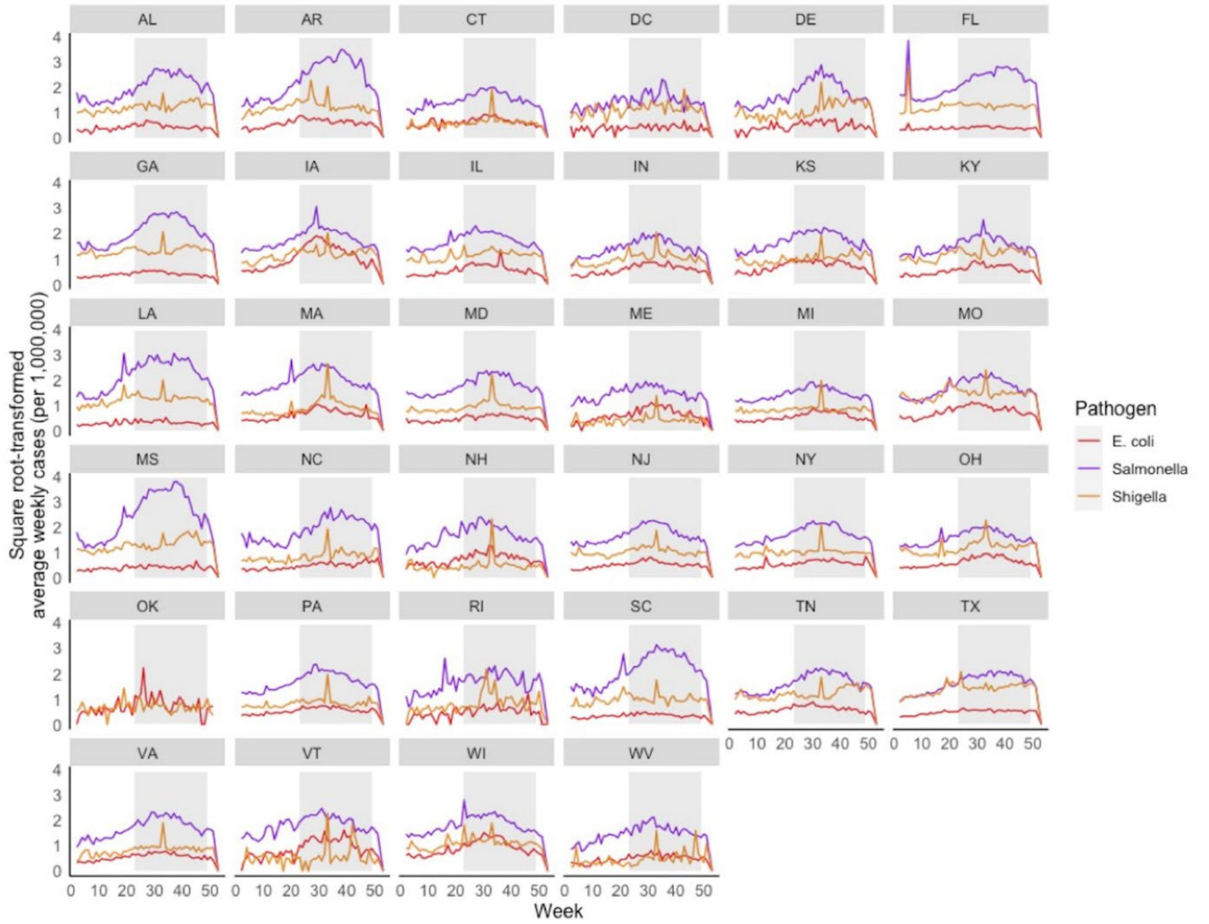

Appendix Figure 2. Average weekly cases of *Escherichia coli* infection, salmonellosis, and shigellosis per 1,000,000 population by state in a study of waterborne infectious diseases associated with exposure to tropical cyclonic storms, United States, 1996–2018. Average weekly cases were square-root transformed for infectious disease surveillance data in the National Notifiable Diseases Surveillance System (https://www.cdc.gov/nndss); not all infections were reported for the entire study period (Table 1). The shaded region represents the weeks encompassed in the annual Atlantic storm season, June 1–November 30. AL, Alabama; AR, Arkansas; CT, Connecticut; DC, Washington, DC; DE, Delaware; FL, Florida; GA, Georgia; IL, Illinois; IN, Indiana; KY, Kentucky; LA, Louisiana; MA, Massachusetts; MD, Maryland; ME, Maine; MI, Michigan; MS, Mississippi; NC, North Carolina; NH, New Hampshire; NJ, New Jersey: NY, New York; OH, Ohio; OK, Oklahoma; PA, Pennsylvania; RI, Rhode Island; SC, South Carolina; TN, Tennessee; TX, Texas; VA, Virginia; VT, Vermont; WI, Wisconsin; WV, West Virginia.

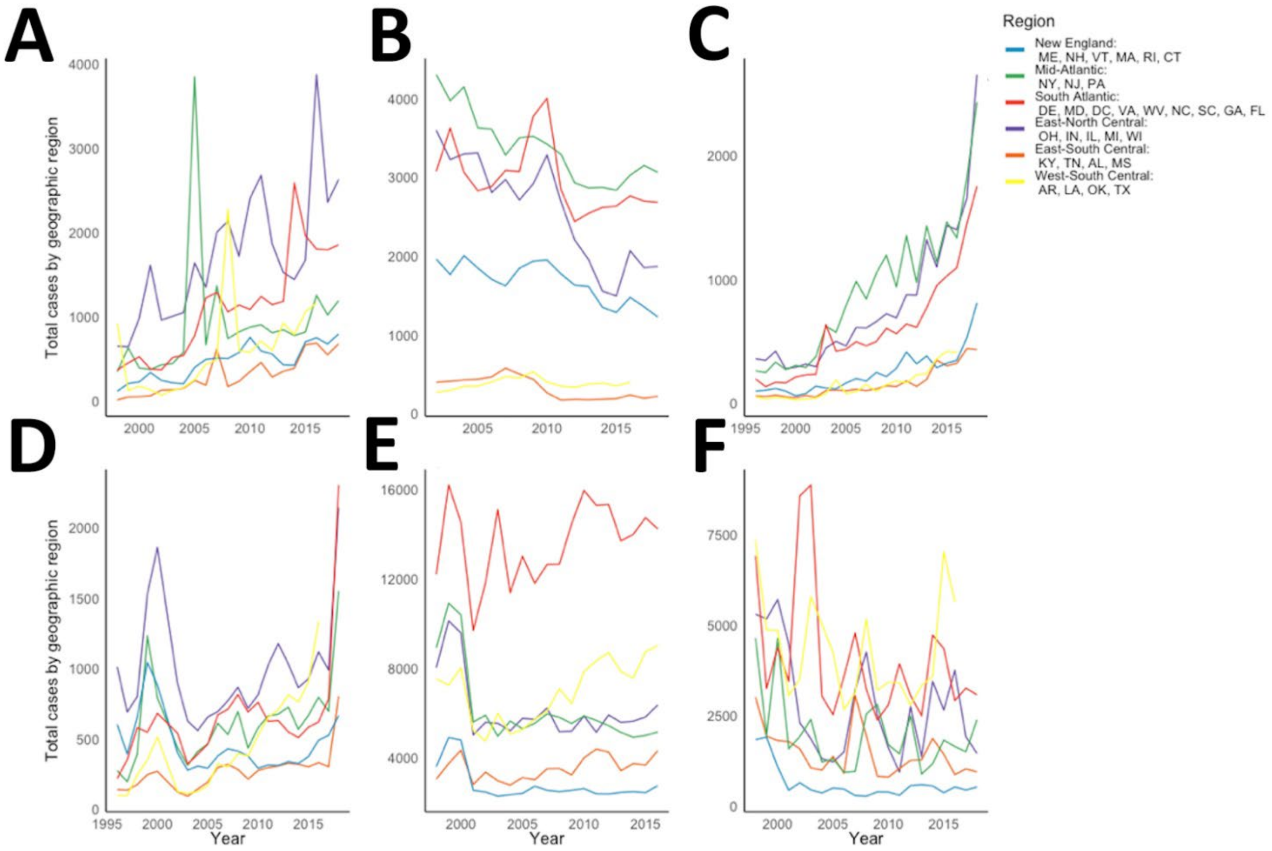

Appendix Figure 3. Total weekly cases by geographic region reported to the National Notifiable Diseases Surveillance System (https://www.cdc.gov/nndss), United States, 1996–2018. Data are included for the following waterborne diseases: A) cryptosporidiosis; B) giardiasis; C) Legionnaires' disease; D) Escherichia coli infection; E) salmonellosis; and F) shigellosis. Not all infections were reported for the entire study period (Table 1). AL, Alabama; AR, Arkansas; CT, Connecticut; DC, Washington, DC; DE, Delaware; FL, Florida; GA, Georgia; IL, Illinois; IN, Indiana; KY, Kentucky; LA, Louisiana; MA, Massachusetts; MD, Maryland; ME, Maine; MI, Michigan; MS, Mississippi; NC, North Carolina; NH, New Hampshire; NJ, New Jersey: NY, New York; OH, Ohio; OK, Oklahoma; PA, Pennsylvania; RI, Rhode Island; SC, South Carolina; TN, Tennessee; TX, Texas; VA, Virginia; VT, Vermont; WI, Wisconsin; WV, West Virginia.

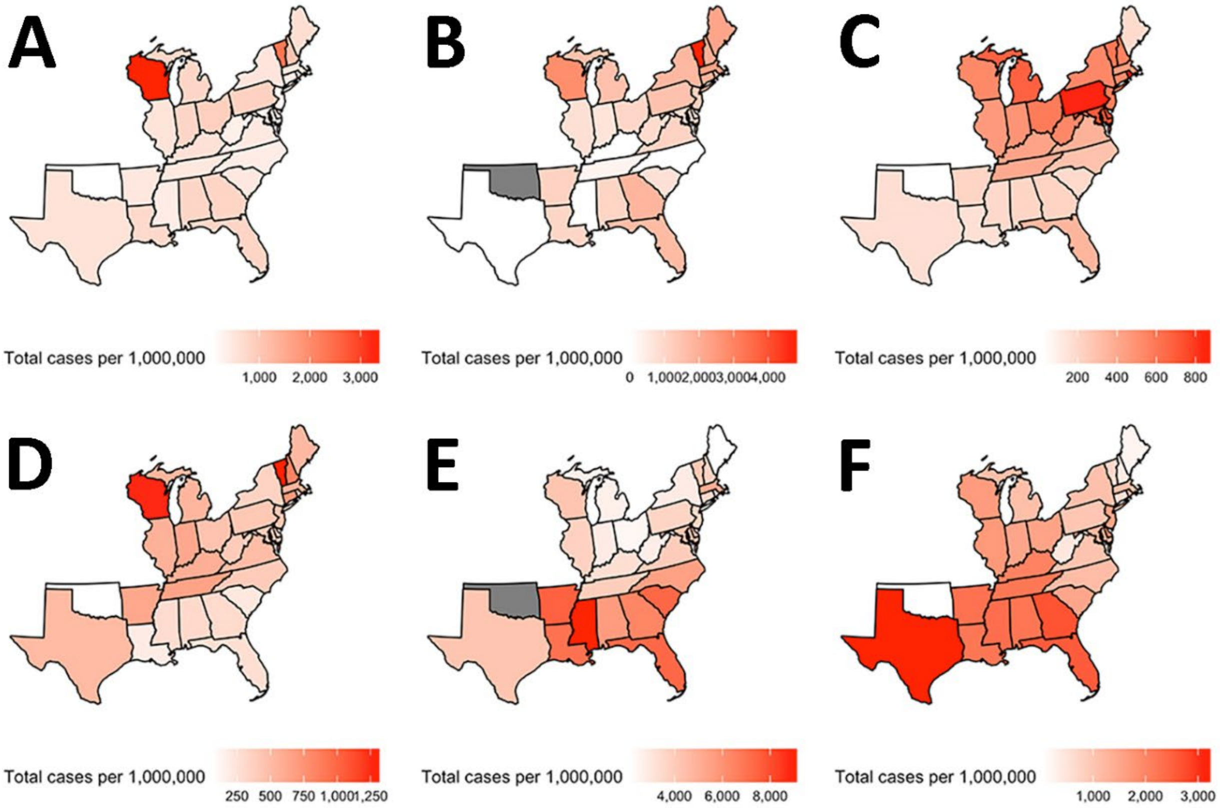

Appendix Figure 4. Cumulative cases per 1,000,000 population in each US state during 1996–2018 for each infectious disease in in the National Notifiable Diseases Surveillance System (NNDSS; https://www.cdc.gov/nndss). Data are included for the following waterborne diseases: A) cryptosporidiosis; B) giardiasis; C) Legionnaires' disease; D) Escherichia coli infection; E) salmonellosis; and F) shigellosis. Gray states represent those that did not contribute to NNDSS for a given infectious disease; not all infections were reported for the entire study or in each state (Table 1).

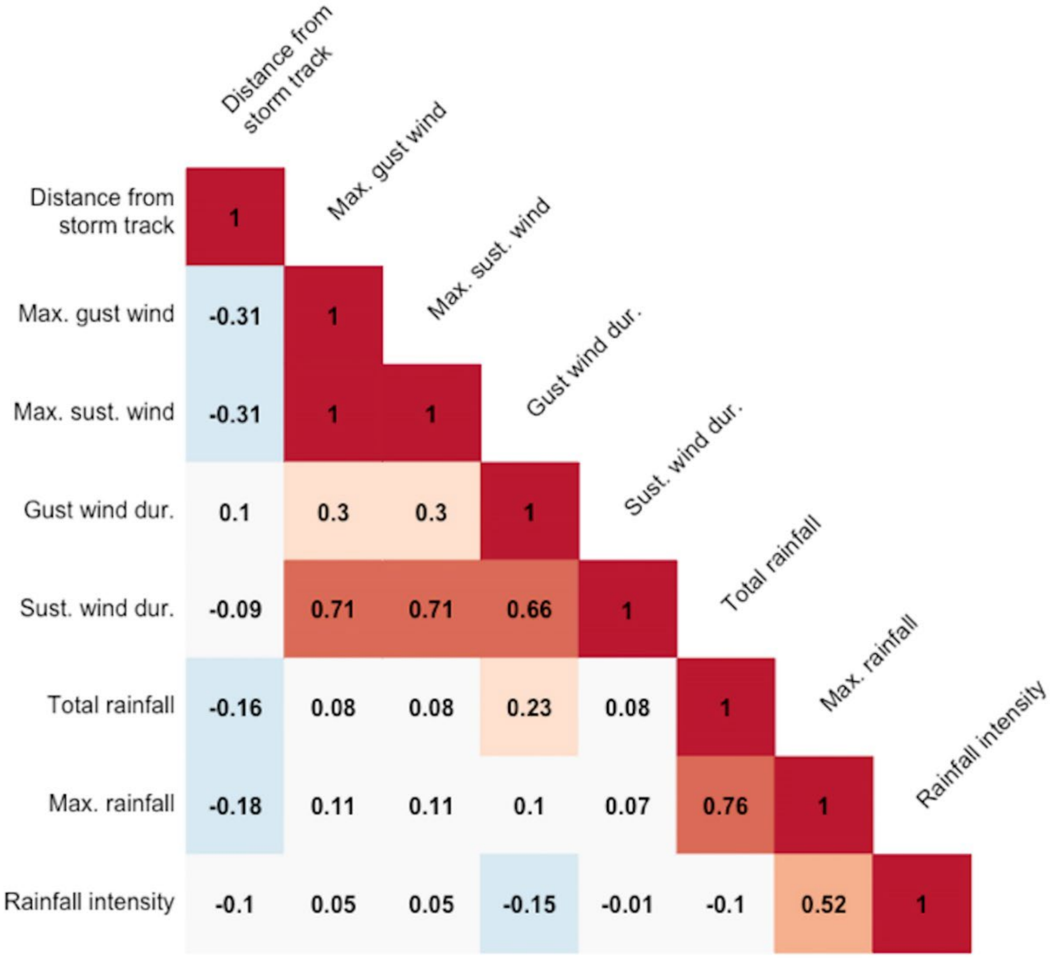

Appendix Figure 5. Correlation matrix for storm characteristics related to rainfall, windspeed, and distance from storm track in a study of waterborne infectious diseases associated with exposure to tropical cyclonic storms, United States, 1996–2018.

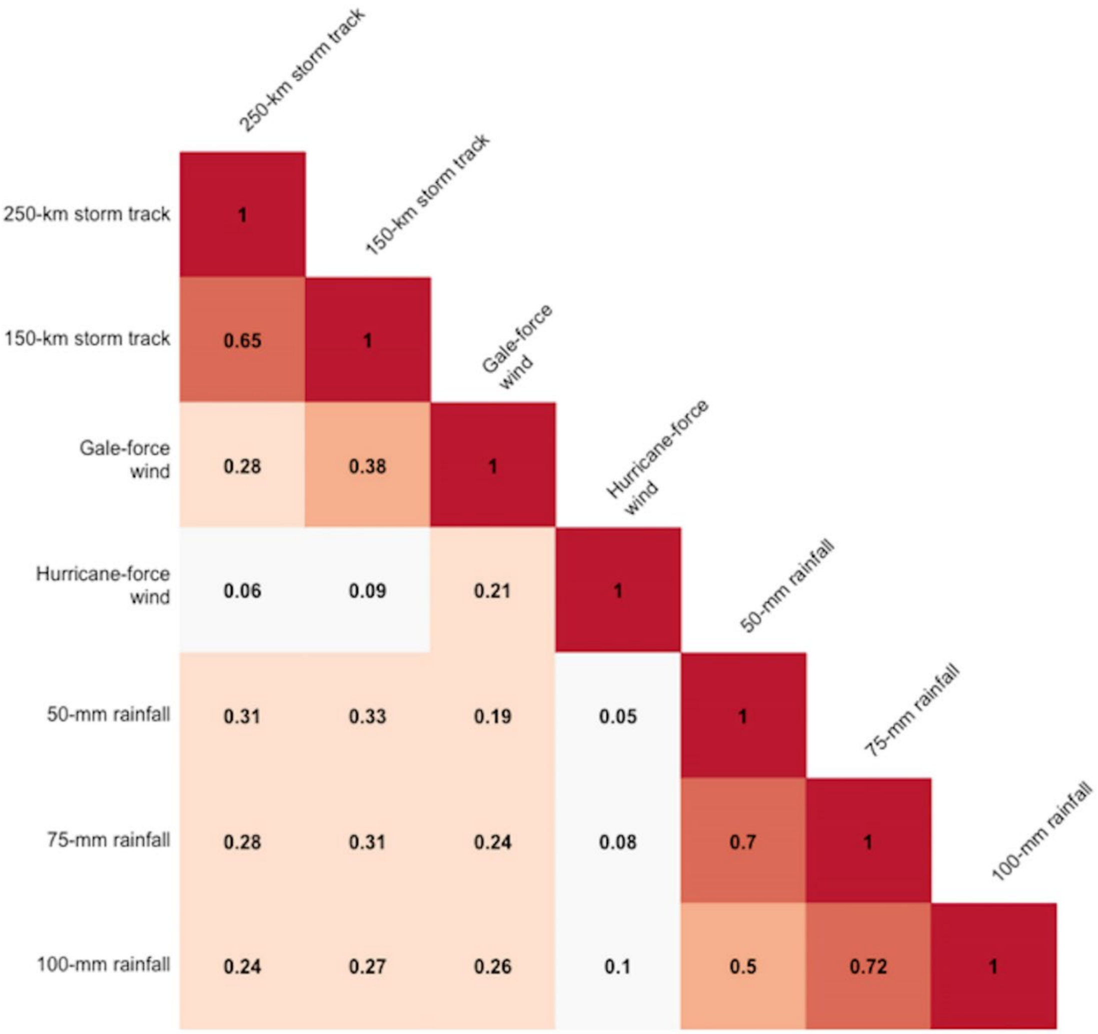

Appendix Figure 6. Correlation matrix for storm exposure thresholds for rainfall, windspeed, and distance from storm track in a study of waterborne infectious diseases associated with exposure to tropical cyclonic storms, United States, 1996–2018.

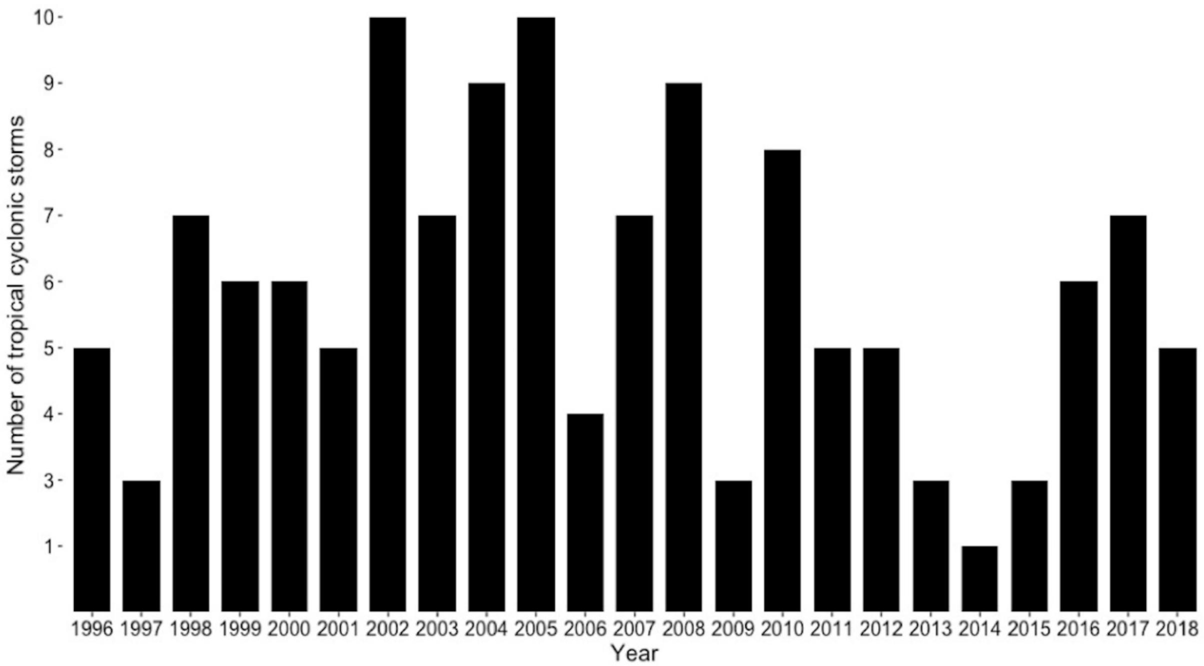

Appendix Figure 7. Number of tropical cyclonic storms that made landfall each year in the United States, 1996–2018.

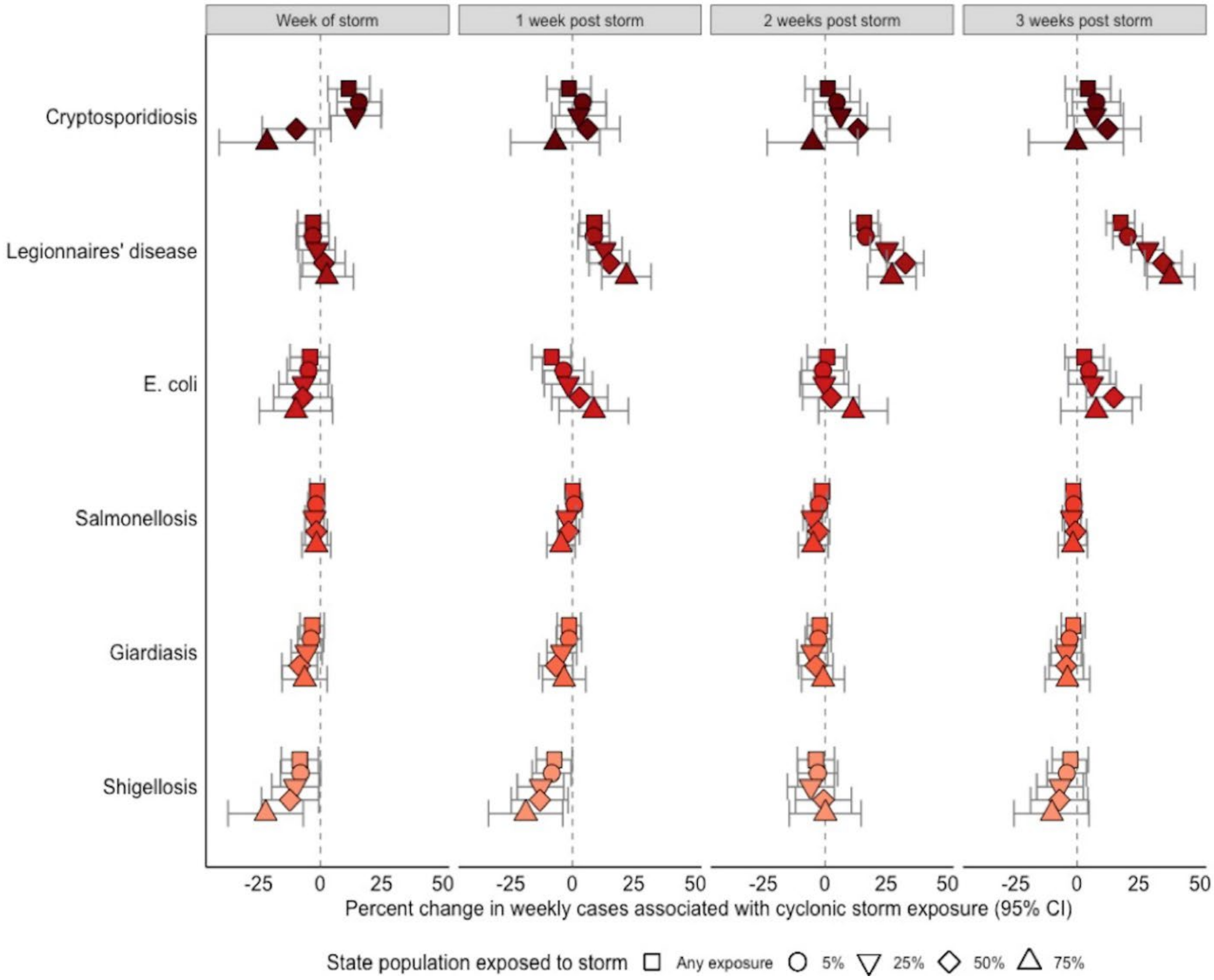

Appendix Figure 8. Average percent change in weekly cases of waterborne infectious diseases associated with exposure to tropical cyclones, United States, 1996–2018. Exposure is defined as being <u><</u>250 km of the storm track. The estimates (shape) and Bonferroni-corrected 95% CIs (bars) are reported for each infectious disease (color) and population-exposure threshold (shape); estimates are reported for week of the storm (week 0) and 1–3 weeks post storm.

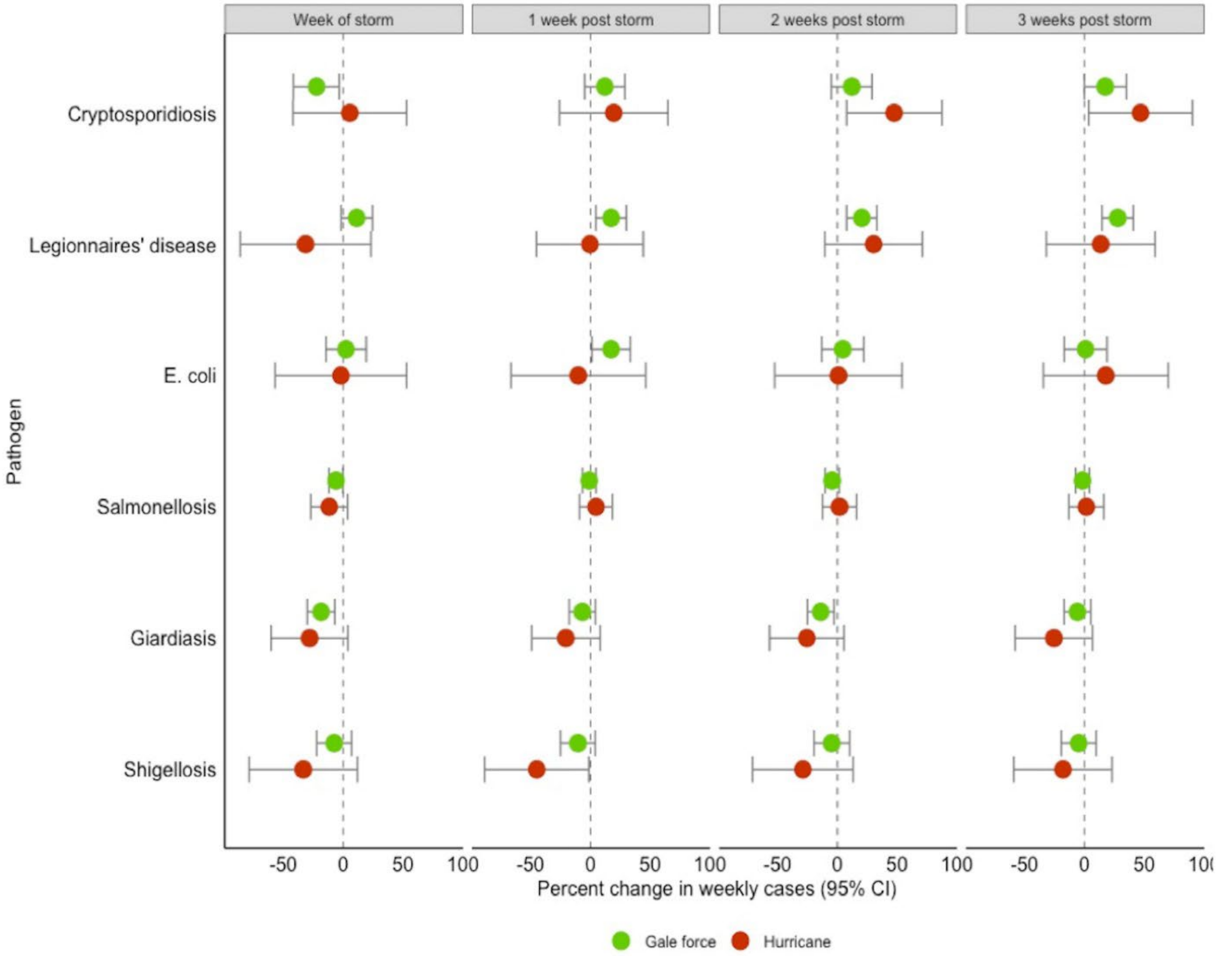

Appendix Figure 9. Average percent change in weekly cases of waterborne infectious diseases associated with exposure to tropical cyclones, United States, 1996–2018. Exposure is defined as sustained gale-force winds, >34 knots (green) or hurricane-force winds, >64 knots (red) and 5% state population in the exposed threshold. The estimates (shape) and Bonferroni-corrected 95% CIs (bars) are reported for each infectious disease (color) and population-exposure threshold (shape); estimates are reported for week of the storm (week 0) and 1–3 weeks post storm.
